# MAKING OLDER MIGRANTS NARRATIVES VISIBLE: AN INTERSECTIONAL ETHICAL ANALYSIS FROM AN AGING STUDIES PERSPECTIVE

**DOI:** 10.1093/geroni/igae098.0879

**Published:** 2024-12-31

**Authors:** Anna-Christina Kainradl

**Affiliations:** University of Graz, Graz, Steiermark, Austria

## Abstract

Despite their heterogeneity, the lives of older migrants are in many ways shaped by intersecting patterns of injustice (Ciobanu et al. 2017). In particular when it comes to health issues, the interplay of race, gender, class, nationality, and age often impedes access and effectiveness of health services for this population group. In order to better address these challenges, it is essential to shift from the dominant perspective of professionals and family members to that of older people with migration biographies (Tezkan-Güntekin 2020). In my paper, I explore findings from an intersectional ethical analysis of the care narratives of older migrants and professionals in the context of aging studies. The paper examines the extent to which the concept of intersectionality as an analytical lens for the narratives of older migrants brings in new perspectives to their negotiations of age(ing). In analyzing the potential of the concept of intersectionality within the aging studies framework, further insights and challenges of postcolonial perspectives for ethical analysis are discussed. This theoretical paper shows how we can further apply and develop the concept of intersectionality in order to better understand the care narratives of older migrants.

